# Inflammatory insults and mental health consequences: does timing matter when it comes to depression?

**DOI:** 10.1017/S0033291716000672

**Published:** 2016-05-16

**Authors:** A. Du Preez, J. Leveson, P. A. Zunszain, C. M. Pariante

**Affiliations:** Department of Psychological Medicine, Stress, Psychiatry and Immunology Laboratory, Institute of Psychiatry, Psychology & Neuroscience, King's College London, London, UK

**Keywords:** Depressive disorder, early life, inflammation.

## Abstract

It has become widely accepted that the immune system, and specifically increased levels of inflammation, play a role in the development of depression. However, not everyone with increased inflammation develops depression, and as with all other diseases, there are risk factors that may contribute to an increased vulnerability in certain individuals. One such risk factor could be the timing of an inflammatory exposure. Here, using a combination of PubMed, EMBASE, Ovid Medline and PsycINFO, we systematically reviewed whether exposure to medically related inflammation *in utero*, in childhood, and in adolescence, increases the risk for depression in adulthood. Moreover, we tried to determine whether there was sufficient evidence to identify a particular time point during the developmental trajectory in which an immune insult could be more damaging. While animal research shows that early life exposure to inflammation increases susceptibility to anxiety- and depressive-like behaviour, human studies surprisingly find little evidence to support the notion that medically related inflammation *in utero* and in adolescence contributes to an increased risk of developing depression in later life. However, we did find an association between childhood inflammation and later life depression, with most studies reporting a significantly increased risk of depression in adults who were exposed to inflammation as children. More robust clinical research, measuring direct markers of inflammation throughout the life course, is greatly needed to expand on, and definitively address, the important research questions raised in this review.

## Introduction

One of the most important developments in translational mental health is the observation that the inflammatory system is involved in the pathogenesis of major depressive disorder (MDD) (Bufalino *et al.* 2013; Valkanova *et al.* 2013; Zunszain *et al.* 2013; Kiecolt-Glaser *et al.* 2015; Miller & Raison, 2015). Activation of the immune system in subsets of depressed patients plays a role not only in disease progression, but also in determining the success of antidepressant therapy (Zunszain *et al.* 2011, 2013; Haroon *et al.* 2012; Strawbridge *et al.* 2015). However, the temporal relationship remains largely unclear: is increased inflammation a cause or an effect of MDD?

Interestingly, exposure to increased inflammation may indeed play a causal role in the pathogenesis of depression, as shown from research detailing how a high proportion of patients undergoing treatment with interferon (IFN)-*α*, a cytokine used for cancer or viral hepatitis C, go on to develop depression (Capuron *et al.* 2001; Bonaccorso *et al.* 2002; Horikawa *et al.* 2003; Loftis & Hauser, 2004; Raison *et al.* 2005). Furthermore, various longitudinal studies support that diabetes mellitus, obesity, and cardiovascular disease, all characterized as low-grade chronic inflammatory states, are significant predictors of depression in later life (Mezuk *et al.* 2008; Nabi *et al.* 2010; Hare *et al.* 2014; Luppino *et al.* 2015).

However, not everyone exposed to increased inflammation develops depression. As with all other diseases, there are risk factors that contribute to an increased vulnerability in certain individuals. Such risk factors could include the type, severity, frequency and/or the timing of an inflammatory challenge. Indeed, research has already shown that the timing and/or age at exposure may be an important predictor of future psychopathology, with exposure to adverse experiences particularly in early life being consistently associated with increased susceptibility to a variety of neuropsychiatric disorders (Turecki *et al.* 2014; Visser *et al.* 2014; Kalmakis & Chandler, 2015; Trotta *et al.* 2015). In two prospective studies, although depression in adulthood was associated with an accumulation of stressors across the life course, most originated in the first years of life (Clark *et al.* 2010; Colman *et al.* 2014). Furthermore, childhood adversity was either directly associated with adolescent, early adulthood, and mid-life affective disorder psychopathology (Clark *et al.* 2010), or was associated with intermediate risk factors that subsequently increased the risk of future depression (Colman *et al.* 2014). Thus, it would seem that adversity in early life has important effects on the life course of depression, and that timing of such adversity may be an important factor in the aetiology of the disorder.

Admittedly, there may even be a time point in early life when individuals are most vulnerable to adverse exposure. In 2012, Bosch and colleagues demonstrated how the sequelae of early life adversity depended on the age at the time of exposure, showing how timing of an adverse event could differentially alter the functioning of the hypothalamic-pituitary-adrenal (HPA) axis in later life. Specifically, they showed how hypercortisolism was a potential consequence of adverse exposure between 6 and 11 years, while adverse exposure between 12 and 15 years contributed to hypocortisolism. Interestingly, individuals exposed before the age of five had no alterations in stress responsivity (Bosch *et al.* 2012). This suggests that the first 5 years of life could represent a stress-hypo-responsive period (Sapolsky & Meaney, 1986) – a temporary and well-documented developmental period in life characterized by attenuated stress responsivity (Gunnar *et al.* 1996; Larson *et al.* 1998; Gunnar & Donzella, 2002) – the evolutionary purpose of which is thought to promote maternal-infant attachment, with low levels of cortisol attributed to maintaining and reinforcing this attachment (Moriceau & Sullivan, 2007). Poignantly, impaired maternal-infant attachment has been associated with poor emotional outcomes in offspring (Leckman-Westin *et al.* 2009), so further understanding of this physiological phenomenon could help extend our knowledge on the aetiology of depression. Indeed, the idea that the stress system (and perhaps even the immune system) may be biologically more or less responsive depending on developmental age could explain why certain types of exposures at certain times in life may exert differential outcomes, and this study emphasizes not only the importance of the type of adversity and the frequency of its occurrence, but also the timing of exposure.

To date, no clinical study has established whether there exists a critical period in life when adversity in the form of a medically-related immune insult may increase one's susceptibility to mental illness. However, one of the hallmarks of the developing immune system is that it exhibits an increased sensitivity for environmentally induced toxicity compared to the fully matured immune system of an adult (Dietert *et al.* 2000; Holladay & Smialowicz, 2000; Dietert, 2013). Moreover, an early life inflammatory insult can result in the impairment of a variety of biological systems involved in the aetiology of MDD, including the neuroendocrine system (Rivest, 2010). Indeed, it seems highly plausible that a ‘critical window’ of vulnerability exists for immune system activation on mental health susceptibility, and that this window of vulnerability could exist when the immune system is still maturing in early life, which starts *in utero,* and continues until the age of 15 years (Hannet *et al.* 1992; Osugi *et al.* 1995; Holt & Jones, 2000).

### Review objectives

The primary objective of this article is to review the current clinical literature in order to elucidate whether exposure to an inflammatory insult early in life, driven by medical or infective causes, increases the risk for depression later in life. Given that neural development extends from the embryonic period through to adolescence (Rice & Barone, 2000; Johnson, 2001), and that this coincides with the development of the immune system (Hannet *et al.* 1992; Osugi *et al.* 1995; Holt & Jones, 2000) we focus on studies reporting exposure to increased inflammation in three developmental life stages: antenatal, childhood (birth to age 12), and adolescence (age 13–18). We specifically exclude studies where inflammation was driven by exposure to psychosocial stress or adversity. Being able to identify when during the developmental trajectory an immune insult is more detrimental could have significant implications in terms of developing successful prevention strategies, raising awareness, and targeting more vulnerable individuals, and as such, it is important to establish whether timing does indeed matter.

## Method

### Search strategy and limits

A combination of PubMed, EMBASE, Ovid Medline and PsycINFO databases were used to systematically select studies for discussion, and reference lists of selected papers were manually searched to check for any additional studies. An independent systematic search of all aforementioned databases was carried for each life stage under study.

We included only clinical studies that were longitudinal in nature, and where there was a minimum of 2 years between exposure and mental health assessment, thereby minimizing any potential contamination of measures. No publication date restrictions were imposed, but our searches were limited to English-language studies only.

### Inflammatory exposure: definition and limits

We defined an inflammatory challenge as any illness pertaining to increased inflammation, which we classified as either a direct, or indirect, immune challenge.

A direct immune challenge was characterized as any bacterial, fungal, parasitic, allergenic and/or viral infection/illness, either chronic or acute, in which the primary host's response to infection/illness was inflammation. Chronic illnesses resulting in the sustained use of immunosuppressant medication, or any autoimmune disease/condition resulting in organ transplantation were excluded.

An indirect immune challenge was defined as any exposure to an illness/condition characterized as a systemic inflammatory state. Substantial evidence chronicles the activation of the immune system in diabetes mellitus (gestational, types I and II) (Donath & Shoelson, 2011; Calle & Fernandez, 2012), obesity (Kredel & Siegmund, 2014; de Jong *et al.* 2014), congenital heart disease (Sharma *et al.* 2003; Allan *et al.* 2010), and cardiovascular disease (Hansson, 2005; Mangge, 2014). Reciprocity between inflammation and these conditions have been consistently demonstrated, such that they are typically referred to as chronic low-grade inflammatory states. As such, we included all studies reporting (*a*) exposure to maternal obesity and diabetes antenatally (*in utero*), and (*b*) living with diabetes, cardiovascular disease, and/or obesity postnatally, in relation to the development of depression in later life. Although cancer is a well-known inflammatory state (Payne, 2014; Roxburgh & McMillan, 2014), due to the vast heterogeneity of this disease, studies on cancer were excluded.

### Depressive psychopathology: definition and limits

Depressive psychopathology was conceptualized by the use of affective/mood symptoms and diagnoses. Most studies assessed depression and/or depressive symptomology in adult participants, i.e. aged >18 years, but we also included papers reporting symptoms or diagnoses of depression in adolescent participants, as the effect(s) of early-life adversity on mental health may have emerged by this time point (Pawlby *et al.* 2011, Plant *et al.* 2015). For studies assessing mental health in adolescence, we also accepted papers reporting symptoms of internalizing and externalizing behaviours, which have been shown to predict adulthood affective disorders (Roza *et al.* 2003; Clark *et al.* 2010).

For a full list of key words used in our searches see Supplementary online Appendix.

## Results

**Fig. 1.**
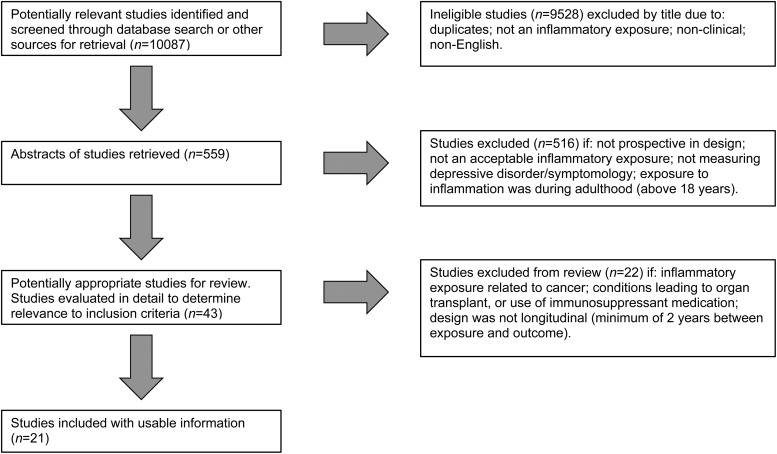
Flow diagram depicting the search strategy employed, the number of studies identified at each stage, the number of studies excluded at each stage and those included in the review.

Fig. 1 highlights the number of studies identified at each stage of our search strategy for all stages combined. Of the 10 087 papers initially flagged up, only 22 clinical studies were eligible and ultimately included in our review.

### Exposure to increased inflammation antenatally

Table 1 displays all clinical studies that directly and indirectly investigated the effect of an antenatal inflammatory challenge on depression susceptibility in later life.

**Table 1.** Studies examining the association between in utero exposure to inflammation and risk for depression in later life

Study	Objectives	Sample/design	Inflammatory exposure	Timing of exposure	Psychopathological outcome	Time of outcome	Findings
**Direct inflammatory insult**							
Brown *et al*. 1995	To examine whether exposure to an influenza epidemic in Holland would increase the risk for adult affective disorders	980697 participants (193 701 exposed)	Potential exposure to A2 influenza virus	Antenatally	Major depressive disorder	23–31 years	–^a^
		Ecological Study	Those that were *in utero* from Sept. 1957 to July 1958		Data on all hospital admissions for affective disorders were provided by the Dutch psychiatric registry, based on International Classification of Disease (ICD-9) diagnosis		
Machón *et al.* 1997	To determine whether exposure to an influenza epidemic in Finland would increase the risk for adult major affective disorder	1378 participants (163 exposed)	Potential exposure to A2/Singapore influenza virus	Antenatally	Major depressive disorder	> 30 years	–^b^
		Ecological study	All adults born from Nov. 1957, to Aug. 1958 with diagnosis of depression		Based on World Health Organization classification system, using ICD-8		
Mino *et al*. 2000	To examine the relationship between the birth of patients with mood disorders and influenza epidemics in Japan	361 participants (61 exposed)	Potential exposure to 2 strains of influenza virus: A2 Asian, and AB mixed type influenza	Antenatally	Depressive disorder	> 30 years	–^a^
					Diagnosed using ICD-10		
		Ecological study	All adults born 5 months after each wave of influenza: June-July 1957, Nov.-Dec. 1957, Apr.-May 1958, July-Sept. 1962 and 1965				
Mellins *et al*. 2003	To examine the long-term effects of *in utero* exposure to human immunodeficiency virus (HIV)	307 participants (96 HIV-infected)	HIV infection	Antenatally	Emotional and behavioural problems	3–17 years	–^a^
		Prospective study	Confirmed by routine clinical and laboratory/virology evaluations		Conner's Parent Rating Scale (CPRS), parental report		
Gaughan *et al*. 2004	To examine the long-term effects of *in utero* exposure to human immunodeficiency virus (HIV)	2298 HIV-infected and 1021 HIV-exposed, uninfected	HIV infection	Antenatally	Depression	>15 years	–^a^
		Prospective study	Confirmed by routine clinical and laboratory/virology evaluations		Assessed using General Health Assessment for Children. Incidence of psychiatric hospitalizations obtained from the National Hospital Discharge Survey (NHDS)		
Dong Pang *et al*. 2009	To assess whether *in utero* viral infections resulted in increased risk of depression in later life	6152 participants (3076 exposed)	Exposure to range of viral infections *in utero* (e.g. rubella, influenza, mumps, varicella, herpes foster, measles, Hodgkin's, multiple sclerosis, cytomegalovirus)	Antenatally	Depressive disorder Diagnosed using ICD-9	33–34 years	–^a^
		Prospective study					
			Assessed by primary-care physician. Information collated using morbidity questionnaire				
**Indirect inflammatory insult:** No studies identified							

a No differences in risk of depression.

b Significantly increased risk of depression.

#### Exposure to maternal infection *in utero*

Although several studies have associated maternal infections with adult psychiatric disorders, predominantly in schizophrenia, autism spectrum disorder, epilepsy and cerebral palsy (Knuesel *et al.* 2014), the impact of maternal infection on depression in offspring is less well-known. To date, only six clinical papers examined whether exposure to infection during pregnancy increased risk of offspring depression (Brown *et al.* 1995; Machón *et al.* 1997; Mino *et al.* 2000; Mellins *et al.* 2003; Gaughan *et al.* 2004; Pang *et al.* 2009).

In one such study, a significant increase in depression was reported for individuals exposed during their second trimester to a Finnish influenza epidemic compared to control subjects born 6 years prior to the outbreak (13% *v*. 2%) (Machón *et al.* 1997). In contrast, two studies examining patients with mood disorders born during an influenza epidemic in Holland and Japan found no significant differences in the risk for depression in patients born during the epidemics (Brown *et al.* 1995; Mino *et al.* 2000). However, the contradictory findings between these studies could partially be explained by other environmental factors such as diet, and indeed, low incidence rates of inflammatory disease in Greenland Inuit and Japanese people, thought to be attributed to the large consumption of fish containing omega-3 fatty acids, has previously been reported (Simopoulos, 2002). Thus, it seems plausible that women in the Japanese cohort may have had diets rich in omega-3 fatty acids, which consequently may have bestowed some protection against depression (Lin & Su, 2007; Sublette *et al.* 2011; Martins *et al.* 2012).

Recently, however, a larger UK study examining the effect of a variety of prenatal viral infections and offspring depression found no overall increased risk for depression associated with viral exposure (Pang *et al.* 2009). Similarly, two large studies investigating the impact of antenatal exposure to human immunodeficiency virus (HIV) on the subsequent development of depression in later life found that HIV exposure did not predict the development of poor emotional and/or behavioural outcomes. Although a high prevalence of behavioural problems did exist among HIV-infected children, these studies found that neither HIV infection nor prenatal drug exposure was the underlying cause (Mellins *et al.* 2003; Gaughan *et al.* 2004).

#### Exposure to gestational diabetes and obesity *in utero*

Interestingly, no studies on the effect of gestational diabetes or obesity on increased risk of offspring depression were found. Although gestational diabetes has been linked to altered brain development and behaviour in offspring, specifically in autism spectrum disorder (Xu *et al.* 2014) and attention-deficit hyperactivity disorder (Nomura *et al.* 2012), no study was found in relation to depression.

### Exposure to increased inflammation in childhood

Clinical studies examining whether direct or indirect exposure to inflammation in childhood predicts depression in later life are given in Table 2.

**Table 2.** Studies examining the association between childhood exposure to inflammation and risk for depression in later life

Study	Objectives	Sample/design	Inflammatory exposure	Timing of exposure	Psychopathological outcome	Time of outcome	Findings
**Direct inflammatory insult**							
Gaughan *et al*. 2004	To examine the long-term effects of postnatal exposure to human immunodeficiency virus (HIV)	2298 HIV-infected and 1021 HIV-exposed, uninfected	HIV infection	Median age 10 years	Depression	>15 years	–^a^
		Prospective study	Confirmed by routine clinical and laboratory/virology evaluations		Assessed using General Health Assessment for Children. Incidence of psychiatric hospitalizations obtained from the National Hospital Discharge Survey (NHDS)		
Cohen *et al*. 1998	To assess the association between early life illness and mental health in later life	774 participants (233 chronically ill)	Used a checklist of chronic conditions (e.g. heart problems, chronic respiratory conditions, chronic pain, orthopaedic problems)	1–10 years	Depressive disorder	At 13, 16 and 22 years	–^b^
		Prospective study	Measured by both self, and mothers, report		Diagnosis determined by the Diagnostic Interview Schedule for Children (DISC)		
Packham *et al*. 2002	To investigate the effect of juvenile idiopathic arthritis (JIA) on mental health in later life.	246 participants with JIA (no control group)	Meeting standard criteria for JIA	0–16 years	Depression	18–71 years	–^b^
		Prospective study	Measured by interview, clinical examination and notes review by the same rheumatologist		Measured with the Hospital Anxiety and Depression (HAD) scale		
Goodwin, 2011	To determine the association between bacterial infection in early life and mental health in a community sample	1285 participants (14 with infection)	A severe infection needing antibiotics	0–1 year	Depressive disorder	9–17 years	–^b^
		Retrospective design	Measured by parental report		Diagnosis determined using the DISC		
Khandaker *et al*. 2014	To test whether higher serum levels of IL-6 and CRP would increase future risk for depression	4415 participants	Serum levels of IL-6 and CRP	9 years	Depression	18 years	–^b^
		Prospective study	Measured in non-fasting blood samples		Diagnosis determined by Clinical Interview Schedule-Revised (CIS-R) and Mood and Feelings Questionnaire (MFQ)		
Ferro & Boyle, 2015	To evaluate the impact of chronic physical illness on depression and anxiety	10 646 participants (1932 with illness)	Asthma, cerebral palsy, epilepsy, heart condition, kidney condition, any other long-term condition	0–11 years	Anxiety and Depression	14–15 years	–^b^
		Prospective study			Ontario Child Health Study Checklist		
Pless *et al*. 1989	To investigate the effect of chronic physical illness on mental health in later life.	5362 participants (467 chronically ill)	Any physical, non-fatal condition lasting less than 3 months in a given year. Repeated episodes of acute physical illness excluded	<15 years	Emotional disturbance, using self- report	26 years	–^c^
		Prospective study	Measured by parental report and cross-referenced with hospital records		Affective state, using Present State Examination	36 years	
Kokkonen & Kokkonen, 1993	To determine whether chronic physical illness in childhood increases the risk for later life depression	530 participants (407 chronically ill, age-matched to 123 controls)	Chronic disorders: asthma, diabetes, epilepsy, growth hormone deficiency, motor handicaps, rheumatoid arthritis, congenital heart disease	Childhood, age range not defined	Depression	18–25 years	–^c^
		Prospective study			Assessed by Present State Examination		
**Indirect inflammatory insult**							
Kovacs *et al*. 1997	To determine prevalence rates and risk factors for psychiatric disorders associated with type 1 diabetes mellitus	92 participants with diabetes mellitus (no control group)	Diagnosis of classic, acute-onset type 1 diabetes mellitus	8–13 years	Depressive disorder	Median age 20 years	–^b^
		Longitudinal, naturalistic design			Assessed using standardized, semi- structured, symptom-based Interview Schedule for Children and Adolescents (ISCA)		
Areias *et al*. 2013	To test for the effects of different demographic, clinical and psychosocial variables on psychiatric morbidity of participants with congenital heart disease (CHD).	150 CHD patients (no appropriate control group)	CHD. Identified through the paediatric cardiology or adult cardiology outpatient clinic	0–18 years	Major depressive disorder	26 years	–^c^
		Retrospective design		Only 10 participants diagnosed in adolescence	Assessed using schedule for affective disorders and schizophrenia (SADS-L) interview, YSR (Youth Self-Report) and ASR (Adult Self- Report) to assess recent behavioural and emotional problems		
Lašaitė *et al*. 2015	To compare mood state profiles in adult patients with childhood-onset and adulthood-onset type 1 diabetes mellitus	214 participants with diabetes mellitus (no control group)	Diagnosis of classic, acute-onset type 1 diabetes mellitus	0–18 years	Tension-anxiety, depression-dejection	>18 years	–^c^
		Retrospective design	Identified through Lithuanian Diabetes Registry		Evaluated using the Profile of Mood States		

a No differences in risk of depression.

b Significantly increased risk of depression.

c Significantly increased risk of depression for particular subset of cohort.

#### Chronic illness in childhood and depression in later life

Eight studies pertaining to chronic or acute illness in childhood were found (Pless *et al.* 1989; Kokkonen & Kokkonen, 1993; Cohen *et al.* 1998; Packham *et al.*, 2002; Gaughan *et al.* 2004; Goodwin, 2011; Ferro & Boyle, 2015; Khandaker *et al.* 2014). Of these, five used a broad definition of infection/illness, and as such had heterogeneous diagnostic groups, while the other two referred to a specific infection/illness.

#### Exposure to heterogeneously defined physical illness in childhood

One British birth cohort investigated the effect of chronic illness in childhood on the mental health wellbeing of participants at ages 26 and 36 years (Pless *et al.* 1989). The authors found no overall significantly increased reports of psychiatric disorders in chronically ill children compared to healthy controls (men: 6.1% *v.* 3.8%; women: 14.1% *v.* 9%). However, they did find that participants who were ill in childhood and again after 21 years of age were significantly more likely to have a psychiatric disorder compared to other members of the cohort. Additionally, when cohort members were re-interviewed at 36 years of age, specifically to assess current affective state, women who experienced childhood chronic illness had significantly higher depressive scores compared to controls. This study highlights that (*a*) repeated exposure to medically-related inflammation, across two life stages, may be necessary for eliciting psychopathology in some individuals, and (*b*) the effect of early life illness may operate only for a subset of individuals, with women, in this instance, being more vulnerable.

In a second study, the prevalence rates of mental disorders in adults who suffered from a variety of chronic physical illnesses in childhood were compared to age-matched controls (Kokkonen & Kokkonen, 1993). Similar to findings from Pless and colleagues, this study found no significant difference in the prevalence rates of all types of depression in young adults with childhood illness compared to healthy controls (13% *v.* 12%). However, *severe* depression was significantly more common in patients exposed to childhood illness than in controls (6% *v.* 2%). Again, we find only a subset of vulnerable individuals, and, as in this case, unless studies examine diagnosis by severity, participants with milder forms of depression may mask the association between early life illness and more severe forms of depression.

Contrary to the findings of the two aforementioned studies, which all demonstrate an increased vulnerability to depression in only a subset of participants suffering from illness as children, three more recent studies find an overall significant association between childhood infection and the mental health of their cohort (Cohen *et al.* 1998; Goodwin, 2011; Ferro & Boyle, 2015). A large US cohort found that chronic physical illness in childhood predicted an increased risk of future depression in both adolescence [odds ratio (OR) 3.81, 95% confidence interval (CI) 1.55–9.39] and young adulthood (OR 4.04, 95% CI 1.54–10.62) independent of prior depressive episodes and other demographic covariates. Moreover, the authors also showed how immunologically mediated disorders, specifically atopic illness, hay fever and mononucleosis, exhibited strong associations with subsequent onset of depression in both adolescence and young adulthood (Cohen *et al.* 1998). Similarly, another large, but retrospective study, examined the association between infection in the first year of life and mental disorders among youth in a community sample, and reported that early life infection was associated with significantly increased odds of depression (OR 3.7, 95% CI 1.0–13.4) (Goodwin, 2011). However, despite the claim of significantly increased odds of depression, we should be mindful that the confidence intervals included one. Finally, in congruence with both Cohen and colleagues, and Goodwin, another large study found that those chronically ill in childhood reported significantly more symptoms of depression in adolescence compared to healthy controls (OR 2.71 *v.* 2.36) (Ferro & Boyle, 2015).

#### Exposure to specific physical illness in childhood: taking inflammatory markers into account

Thus far there appears to be fairly strong evidence to suggest that exposure to illness in childhood increases the risk for depression in later life, particularly for a subset of individuals. However, none of the aforementioned studies have directly measured levels of inflammation, a concept we should be mindful of in the context of this review. However, there exist three clinical studies that provide some insight into the direct effect of inflammation on depression susceptibility.

One such study, assessing adults with juvenile idiopathic arthritis (JIA), found that patients with systemic-onset JIA had significantly higher levels of depression (10.7%) compared to other JIA subsets. Interestingly, the study also found that depression was most commonly seen when the age of onset was between 6 and 12 years (11.1%) compared to early (2.7%) or late (0%) onset, and that the first episode of depression tended to be between ages 15 and 25 years (38.5%). However, although these findings are highly novel in that they are the first to demonstrate that JIA onset specifically in childhood may exert a greater influence over the subsequent development of depression, the study additionally highlighted how joint inflammation based on the Thompson–Kirwan scale, likely representing the magnitude of systemic inflammation, was not a significant predictor of depression in this cohort (Packham *et al.*, 2002). Similarly, another study examining the long-term effects of postnatal exposure to HIV on mental health in later life found that although HIV infected children were at increased risk for depression compared to healthy controls, immunological and virological markers were not responsible for predicting first admission hospitalizations for depression (Gaughan *et al.* 2004). Interestingly, these studies emphasize how psychological variables, rather than the acute effects of specific inflammatory factors, may explain the majority of variance seen in later life depression.

However, one population-based study showed how participants with increased levels of systemic inflammation in childhood, indicated by higher serum levels of interleukin-6, were at significantly increased risk of developing depression in adulthood (Khandaker *et al.* 2014). Unlike many of the already discussed papers, this study's strength was that it controlled for a wide variety of confounders including past psychological and behavioural problems and maternal psychopathology, potentially controlling for adversity-related causes of inflammation, as well as children with medical conditions. Therefore, these findings are unique in that they show how raised levels of inflammation, not likely accounted for by adversity, infection or illness, can predict future psychopathology. This suggests that perhaps an inherently dysfunctional immune system rather than exposure to inflammation via infection *per se* is the key to understanding the association between inflammation and depression.

#### Living with obesity, diabetes mellitus and congenital heart disease in childhood and later life depression

Widening our search to incorporate studies investigating the long-term impact of childhood obesity, congenital heart disease, and type I diabetes mellitus yielded an additional three papers.

#### Childhood obesity and depression in later life

Although previous studies have posited a link between adiposity and depression, with inflammation playing a key role in the disorder's pathogenesis (Shelton & Miller, 2011), no published clinical studies investigating the impact of childhood obesity on future depression were found. However, this is unsurprising since only recently has the impact of obesity in relation to mental health been under full investigation. Given the time and resources involved in conducting longitudinal studies, insights into the impact of childhood obesity on mental health in later life is likely to emerge in subsequent years.

#### Type I diabetes mellitus and depression in later life

Two clinical studies investigating the effect of diabetes in childhood on affective disorder psychopathology were found, with contradictory conclusions as to when the sensitive period for disease onset may lie. One study reported an increased risk for depressive disorders in early adulthood for childhood-onset diabetic patients (Kovacs *et al.* 1997), while the other found that adulthood onset type I diabetic women reported higher levels of depression than childhood-onset diabetic patients (Lašaitė *et al.* 2015).

#### Congenital heart disease and depression in later life

The influence of congenital heart disease on later life affective disorder psychopathology has been investigated in only one longitudinal study, which found no overall difference in the mental health outcomes of these patients and healthy controls. However, similar to previous findings from other studies, the study did find that for a subset of the cohort – female patients, and those with more complex forms of the disorder – significantly higher levels of anxiety and depression were reported. Moreover, the authors found that age at assessment was important for evaluating the impact of these disorders on later mental health, finding that those aged 19–26 years had more symptoms of anxiety/depression than those aged 12–18 years (Areias *et al.* 2013).

### Exposure to increased inflammation in adolescence

Table 3 displays all clinical studies that have assessed whether direct or indirect exposure to increased inflammation in adolescence predicts future depression.

**Table 3.** Studies examining the association between adolescent exposure to inflammation and risk for depression in later life

Study	Objectives	Sample/design	Inflammatory exposure	Timing of exposure	Psychopathological outcome	Time of outcome	Findings
**Direct inflammatory insult**							
Chen *et al*. 2013	To assess whether allergic rhinitis (AR) increases the risk of depression in later life	8365 participants (1673 with AR)	Diagnosis of AR	12–15 years	Depressive disorder	>22 years	–^b^
		Prospective study	Based on World Health Organization classification system, using the International Classification of Disease (ICD-10)		Based on ICD-9-CM diagnosis		
**Indirect inflammatory insult**							
Jacobson *et al.* 1997	To evaluate the psychological adjustment of young adults with diabetes mellitus	111 participants (57 with diabetes mellitus)	Diagnosis of type 1 diabetes mellitus	9–16 years	Depression	19–26 years	–^a^
		Prospective study			Assessed using the Symptom Checklist-90-Revised (SCL-90R)		
Herva *et al*. 2006	To examine the association between obesity and depression	10 096 participants (377 with obesity)	Obesity classified as a BMI >23.43 kg/m^2^ (males), 23.81 kg/m^2^ (females)	14 years	Depressive disorder	31 years	–^b^
		Prospective study	Self-report		HSCL-25-derpression questionnaire: Self-report		
Anderson *et al.* 2007	To assess whether adolescent obesity isassociated with risk for major depressive disorder (MDD) or anxiety disorder in later life	701 participants (45 with obesity)	Obesity classified as a BMI z score >95th percentile	9–18 years	Depressive disorders	28–39 years	–^c^
		Prospective study	Baseline based on parental report; follow up based on self-report		Assessed using the Structured Clinical Interview for DSM-IV Disorders (SCID-IV)		
Marmorstein *et al.* 2014	To examine prospective associations between obesity from early adolescence and early adulthood and depression	1512 participants	Obesity classified as a BMI >95th percentile	11–24 years	Depressive disorder	14–24 years	–^c^
		Prospective twin study	Height and weight measured using a Detecto mechanical physician scale		Diagnostic Interview Schedule for Children and Adolescents (before 17 years) and the SCID (after 17 years)		
Lašaitė *et al*. 2015	To compare mood state profiles in adult patients with childhood-onset and adulthood-onset type 1 diabetes mellitus	214 participants with diabetes mellitus (no control group)	Diagnosis of classic, acute-onset type 1 diabetes mellitus	0–18 years	Tension-anxiety, depression-dejection	>18 years	–^c^
		Retrospective design			Evaluated using the Profile of Mood States		

a No differences in risk of depression.

b Significantly increased risk of depression.

c Significantly increased risk of depression for particular subset of cohort.

#### Chronic illness in adolescence and depression in later life

A comprehensive search of the literature yielded one study exploring a direct link between adolescent infection/illness and the development of future depression. However, whilst the data for infection during adolescence was lacking, we found one large study evaluating the relationship of allergic rhinitis (AR) to the development of any depressive disorder in later life (Chen *et al.* 2013). In this study, adolescents with AR had a significantly higher prevalence of major depression (2.5% *v.* 1.2%) and any depressive disorder (4.9% *v.* 2.8%) in later life compared to control subjects.

#### Living with obesity, diabetes and cardiovascular disease in adolescence and depression in later life

Thus far only one study pertaining to adolescent direct exposure has been identified, which has limited our ability to identify any patterns. Broadening our search to incorporate studies looking at the effect of indirect inflammatory conditions yielded an additional five papers.

#### Adolescent obesity and depression in later life

Three studies exploring the relationship between obesity in adolescence and the subsequent development of future depression were identified. One study reported an overall positive association between obesity and depressive symptoms in adulthood, finding that a higher body mass index (BMI) at age 14 correlated with higher BMI, leptin, C-reactive protein, and depressive symptoms at age 17. Moreover, the study found that females who were obese in both adolescence and adulthood more frequently reported symptoms of depression (Herva *et al.* 2006). Interestingly, the remaining two studies found no overall association between adolescent obesity and depression, but did find that the increased risk for the development of future depression was gender specific. Anderson and colleagues found that adolescent obesity in females, but not in males, predicted an increased risk for the subsequent development of depression and anxiety disorder (Anderson *et al.* 2007). Similarly, Marmorstein and colleagues reported how only obesity in female adolescents predicted the onset of depression in early adulthood: specifically, it was an onset of obesity after the age of 14 that predicted the development of depression in early adulthood among females in the cohort (Marmorstein *et al.* 2014).

#### Type I diabetes mellitus and depression in later life

With respect to the impact of diabetes on depression susceptibility in later life only two clinical studies were identified. Both studies found no difference in the psychological outcome of adults diagnosed with diabetes in adolescence compared to healthy controls (Jacobson *et al.* 1997; Lašaitė *et al.* 2015). Interestingly, however, Jacobson and colleagues did find that in early adulthood individuals with diabetes had lower self-esteem (Jacobson *et al.* 1997), a considered predisposing factor for depression (Ferro & Boyle, 2015). Therefore, it is possible that these individuals may develop depression in later adulthood.

#### Cardiovascular disease in adolescence and depression in later life

Regarding the effect of cardiovascular disease in adolescence on mental health, no studies were found. However, this was anticipated given that the onset of cardiovascular disease is typically in adulthood.

## Conclusion

### Main findings

We have reviewed all available literature examining the effect of a medically related inflammatory challenge in early life, i.e. antenatally, in childhood and in adolescence, on depression susceptibility in later life. We found no clinical evidence to support that inflammation *in utero* contributes to an increased risk of developing future depression. This was somewhat surprising given that (*a*) animal research consistently supports that increased inflammation neonatally increases depressive-like behaviour in later life (Walker *et al.* 2004, 2006, 2008, 2009; Bilbo *et al.* 2005; Spencer *et al.* 2006, 2011; Galic *et al.* 2008; Roque *et al.* 2014), and (*b*) stress, particularly during pregnancy, is a potential predictor for later life psychopathology (Betts *et al.* 2015; Slykerman *et al.* 2015; Biaggi *et al.* 2016), which is pertinent given the bi-directional relationship between stress and inflammation (Chovatiya & Medzhitov, 2014; Slavich & Irwin, 2014). However, given that cytokines do not cross the placenta barrier in normal term foetuses, this may contribute for our findings (Zaretsky *et al.* 2004; Aaltonen *et al.* 2005).

However, we did find some converging evidence to support that exposure to increased inflammation in childhood increases the risk for adult depression. Furthermore, the evidence suggests that persistent physical health problems in childhood may relate to the presence of greater or more severe psychiatric disorders, and that psychiatric outcome may be gender specific, with females being more vulnerable to exposure. Interestingly, the evidence pertaining to studies assessing exposure to severe conditions in childhood was mixed, but did seem to suggest that for lifelong conditions, such as HIV, diabetes and congenital heart diseases, increased inflammation does not increase susceptibility to depression. However, it is important to note that, for such conditions, treatment strategies may be reducing the overall increased inflammation associated with disease state. Finally, when looking at the direct effect of inflammation in childhood on the susceptibility to depression, the evidence was both limited in quantity and inconsistent in findings, emphasizing the need for further clinical research directly measuring inflammatory markers and evaluating their role in the aetiology of depression.

Looking at adolescent exposure to increased inflammation on future depression risk, a limited quantity of evidence was found. However, we did find some evidence to support that an indirect immune challenge in the form of obesity may increase the risk for future depression, particularly for female adolescents.

### Limitations of the existing literature

It is noteworthy that fewer clinical studies were identified for the antenatal and adolescent life stages, and this made it extremely difficult to establish consistent patterns pertaining to these developmental phases from the available evidence. Although we did find some evidence to support that increased inflammation in childhood, and to some extent in adolescence, increases depression risk in later life, many of these studies did not look specifically at levels of inflammation in their cohorts, and as such we cannot conclude that it was increased inflammation *per se* that predicted depression. Indeed, inflammatory markers were unavailable for the majority of the studies reviewed, predominately because their research question was not addressing the effect of increased inflammation on depression, and as such, this represents a major empirical limitation of conclusions in this review. Other psychological or biological factors associated with these disease states may be accounting for the observed associations.

Furthermore, there are several other limitations that weaken the evidence overall. First, most studies relied on self-reports and/or parental reports of physical illness and affective disorder psychopathology, and it is uncertain whether similar findings would be found with physician-diagnosed medical illnesses. Second, in some studies neither the definition nor timing of physical illness or psychopathology was clearly described. Indeed, several studies assessed exposure to infection across multiple developmental stages, i.e. exposure any time from childhood to early adulthood, and given the large biological and psychosocial differences between the developmental stages, results should be interpreted with caution. Third, important confounders, such as perinatal complications, parental mental health, stress (biological and psychosocial), and childhood maltreatment were not measured and controlled for in analyses in most of the studies. We should be particularly cautious in attributing the observed associations found, especially given the evidence substantiating the involvement of the HPA axis (Pariante & Lightman, 2008), childhood maltreatment (Pawlby *et al.* 2011; Lindert *et al.* 2014; Plant *et al.* 2015), parental psychopathology (McLaughlin *et al.* 2012), and obstetric complications (Räikkönen *et al.* 2008; Tuovinen *et al.* 2010) in the pathogenesis of depression. Fourth, none of the included studies controlled for infections/illnesses across the life course, and it is possible that the increased risk of depression, found in several studies, may be a consequence of an inflammatory insult at another, more vulnerable, later life-stage, or due to the accumulation of inflammatory insults throughout the life course. Therefore, it is difficult to determine whether the immune system may be ‘primed’ to give an enhanced response after repeated inflammatory episodes throughout life. Indeed, elucidating whether an early life infection can ‘prime’ the developing organism's sensitivity to subsequent environmental challenges is one research question that requires investigation in a clinical setting. Finally, several studies lacked an appropriate control group, making drawing firm conclusions tentative, and/or had a small sample size, potentially lacking the required power to thoroughly investigate the research question.

Despite these limitations, there was considerable agreement with regard to many of the findings pertaining to childhood exposure, and consistency was maintained for studies with both large and small sample sizes, and across individuals with different conditions. Moreover, the lack of an overall association between the prevalence rates of depression in the previously ill *v.* healthy participants in some of the other aforementioned studies may have been masked by the fact that participants were no older than 25 years at the time of assessment. Empirical research suggests that the average age of onset for mood disorders is 30 years (Kessler *et al.* 2005), and these studies could have potentially failed to capture the expression of depression in such young cohorts. Furthermore, for all prospective studies, there is a degree of confounding by severity, insofar as the participants with the most severe physical and/or mental disorders may not have been able to meet the demands of the studies in question, and a truly representative outcome may not have been achieved.

### Future work and implications

Investigating the effect of medically-related inflammation at different life stages will ultimately help identify whether the timing of an immune response is relevant to the pathogenesis of depression, and more prospective longitudinal studies that measure depressive outcomes against number and severity of immune activation throughout life is necessary to confirm this link. Inflammatory markers must be measured in order to investigate the association between severity of immune response and the risk of depression developing. Moreover, controlling for important confounders pertaining to stress (e.g. measuring cortisol stress response), as well as parental psychopathology, and adversity throughout the life course is necessary to confidently establish whether there is a particular time point in life where susceptibility to inflammation predisposes individuals to depression.

In conclusion, this review is the first to evaluate the effects of early life inflammation on the development of future depression in a clinical setting. We show that increased inflammation in childhood may increase depression risk in later life, and although more robust clinical research is needed to definitively address the research questions raised by this article, we bring to light that timing may indeed matter. However, we should heed caution before classifying childhood as a potential ‘window of vulnerability’ owing to the vast limitations of the reviewed studies, and the insufficient research pertaining to the other two life stages. Being able to definitively pinpoint when in life individuals are at increased risk from an inflammatory challenge could (*a*) help practitioners and individuals better monitor and report these exposures/risk factors, (*b*) aid early intervention practices, through minimizing and efficiently treating inflammatory conditions during vulnerable stages of development, and (*c*) ultimately tailor treatment plans – all of which are much needed advancements in care practices.

## Supplementary material

For supplementary material accompanying this paper visit http://dx.doi.org/10.1017/S0033291716000672. click here to view supplementary material
