# Supplementary material for: Inflammatory insults and mental health consequences: does timing matter when it comes to depression?
Source: Psychol Med. 2016 May 16;46(10):2041–57. doi: 10.1017/S0033291716000672 (PMC4937234; doi:10.1017/S0033291716000672)
Supplement: Supplementary file 1 [file S0033291716000672sup001.docx]

**SUPPLEMENTARY MATERIAL**

**APPENDIX**

**Search terms**

**Life stage keywords**

**1. *In utero***

antennal(ly)

prenatal(ly)

in utero

pregnancy

pregnant

fetal

**2. *Childhood (0-12 years)***

postnatal(ly)

postpartum

post partum

- childhood(s)
- child hood
- child
- children
- kid(s)
- child youth
- childhood age person
- infant
- infancy
- pre-teen
- pre-pubescent
- peripuberty
- juvenile
- juvenility

**3. *Adolescence (13-18 years)***

adolescence

adolescent

teenager

teen

teens

puberty

pubescence

pubescent

**Exposure and outcome variable keywords**

**1. *Exposure variable***

- ill
- illness(es)
- illness physical

illness chronic

- illness acute

infection(s)

disease(s)

- sick(ness)
- sicknesses
- health problem(s)
- medical problem(s)
- ailment(s)
- allergy
- allergic
- allergenic
- arthritis
- arthritic
- parasite
- parasitic
- bacteria
- bacterial
- virus
- viral
  1. influenza
  2. flu

diphtheria

- 1. tetanus
  2. polio

tuberculosis

measles

chicken pox

fever

fungus

fungal

asthma

- human immunodeficiency virus (HIV)
- obesity
- obese
- overweight
- body mass index (BMI)
- heart disease/disorder
  - - 1. cardiovascular
- chronic
- congenital
- kawasaki
- diabetes
- diabetes mellitus
- inflammation

cytokine

c-reactive protein

interleukin(s)

chemokine(s)

- immune (system) activation
- inflammatory condition
  - 1. inflammatory disorder
    2. inflammatory insult
    3. inflammatory challenge
    4. immune system impairment

**2. *Outcome variable***

mood disorders

affective disorder

unipolar depression

major depressive disorder

depressive disorder(s)

depressive illness(es)

- depression(s)
- depression emotion
- depressive neurosis
- depressive neuroses
- depressive disorders
- depressive disorder
- disorder(s) depressive
- depressive illness
- anxiety
- anxiety disorder(s)

feeling of sadness

- sad
- sadness
- depressed
- depressed mood
- feeling low
- low mood
- low spirits
- despair
- malaise
- melancholic
- melancholy
- miserable
- morose mood
- morosity
- neurosis depressive
- neuroses depressive
